# Upregulation of astroglial connexin 30 impairs hippocampal synaptic activity and recognition memory

**DOI:** 10.1371/journal.pbio.3002075

**Published:** 2023-04-11

**Authors:** Eléonore Hardy, Julien Moulard, Augustin Walter, Pascal Ezan, Alexis-Pierre Bemelmans, Franck Mouthon, Mathieu Charvériat, Nathalie Rouach, Armelle Rancillac

**Affiliations:** 1 Neuroglial Interactions in Cerebral Physiology and Pathologies, Center for Interdisciplinary Research in Biology, College de France, CNRS, Inserm, Labex Memolife, Université PSL, Paris, France; 2 Theranexus, Lyon, France; 3 Doctoral School N° 158, Sorbonne Université, Paris, France; 4 Neurodegenerative Diseases Laboratory, Molecular Imaging Research Center, Paris-Saclay University, CEA, CNRS, Fontenay-aux-Roses, France; Duke University Medical Center, UNITED STATES

## Abstract

Astrocytes crucially contribute to synaptic physiology and information processing. One of their key characteristics is to express high levels of connexins (Cxs), the gap junction–forming protein. Among them, Cx30 displays specific properties since it is postnatally expressed and dynamically upregulated by neuronal activity and modulates cognitive processes by shaping synaptic and network activities, as recently shown in knockout mice. However, it remains unknown whether local and selective upregulation of Cx30 in postnatal astrocytes within a physiological range modulates neuronal activities in the hippocampus. We here show in mice that, whereas Cx30 upregulation increases the connectivity of astroglial networks, it decreases spontaneous and evoked synaptic transmission. This effect results from a reduced neuronal excitability and translates into an alteration in the induction of synaptic plasticity and an in vivo impairment in learning processes. Altogether, these results suggest that astroglial networks have a physiologically optimized size to appropriately regulate neuronal functions.

## Abstract 2

Upregulation of connexin 30 (Cx30) in astrocytes increases the connectivity of astroglial networks, but decreases spontaneous and evoked synaptic transmission, altering the induction of synaptic plasticity and in vivo learning processes, and suggesting that astroglial networks may have a physiologically optimized size to appropriately regulate neuronal functions.

## Introduction

Astrocytes are key elements regulating synaptic physiology and, thereby, brain information processing. These glial cells shape neuronal excitability, synaptic transmission, and plasticity [1–4]. Remarkably, astrocytes exhibit the highest expression level of connexins (Cxs), the gap junction channel–forming proteins. When aligned between adjacent cells, these proteins connect their cytoplasm, enabling direct intercellular communication by diffusion and redistribution of small molecules [4–7], with a molecular weight up to 1.5 kDa [8]. This Cx-mediated astroglial network regulates the efficiency of extracellular potassium (K^+^) and glutamate clearance at synapses [2], as well as long-distance trafficking of energy metabolites to fuel active synapses [2,9]. However, Cxs do not only form gap junction channels with other astrocytes. They can also mediate direct exchange with the extracellular space when forming hemichannels [10,11]. In addition, recent studies have reported channel-independent functions of astroglial Cxs, involving, for instance, intracellular signaling, protein interactions, or cell adhesion [12–15].

Two major Cxs are expressed in astrocytes in the mouse brain: Cx43, a key player in brain development and physiology [4,16], and Cx30, expressed after the second postnatal week [17] in an activity-dependent manner, being endogenously upregulated by spontaneous neuronal activity in hippocampal slices (approximately 50%) [18] or by visual experience in vivo in the visual cortex (approximately 70%) [13]. Furthermore, Cx30 is thought to be involved in basic cognitive and behavioral processes, as its expression is upregulated in vivo in mice raised in an enriched environment [19] and is key for the reactivity of mice to novel environments [20] and contextual memory [21].

Yet, whether increased expression in a physiological range of astroglial Cx30 favors or limits neuronal activity and cognitive capabilities remains unknown. By combining in vivo viral injections of Cx30 selectively in astrocytes with electrophysiological recordings and behavioral testing, we here investigated whether and how local and specific upregulation of Cx30 expression in hippocampal astrocytes from the CA1 region impacts astroglial network connectivity, synaptic transmission, plasticity, and memory. We here demonstrate that mice with increased levels of Cx30 in astrocytes display enhanced size of their gap junction–mediated network but reduced spontaneous and evoked hippocampal synaptic transmission at CA1 Schaffer collateral synapses. We further show that increased level of Cx30 reduces intrinsic neuronal excitability and that this translates into an impairment in synaptic plasticity and learning processes.

## Results

### Local and specific upregulation of Cx30 expression in hippocampal CA1 astrocytes by viral transduction

**Fig 1:**
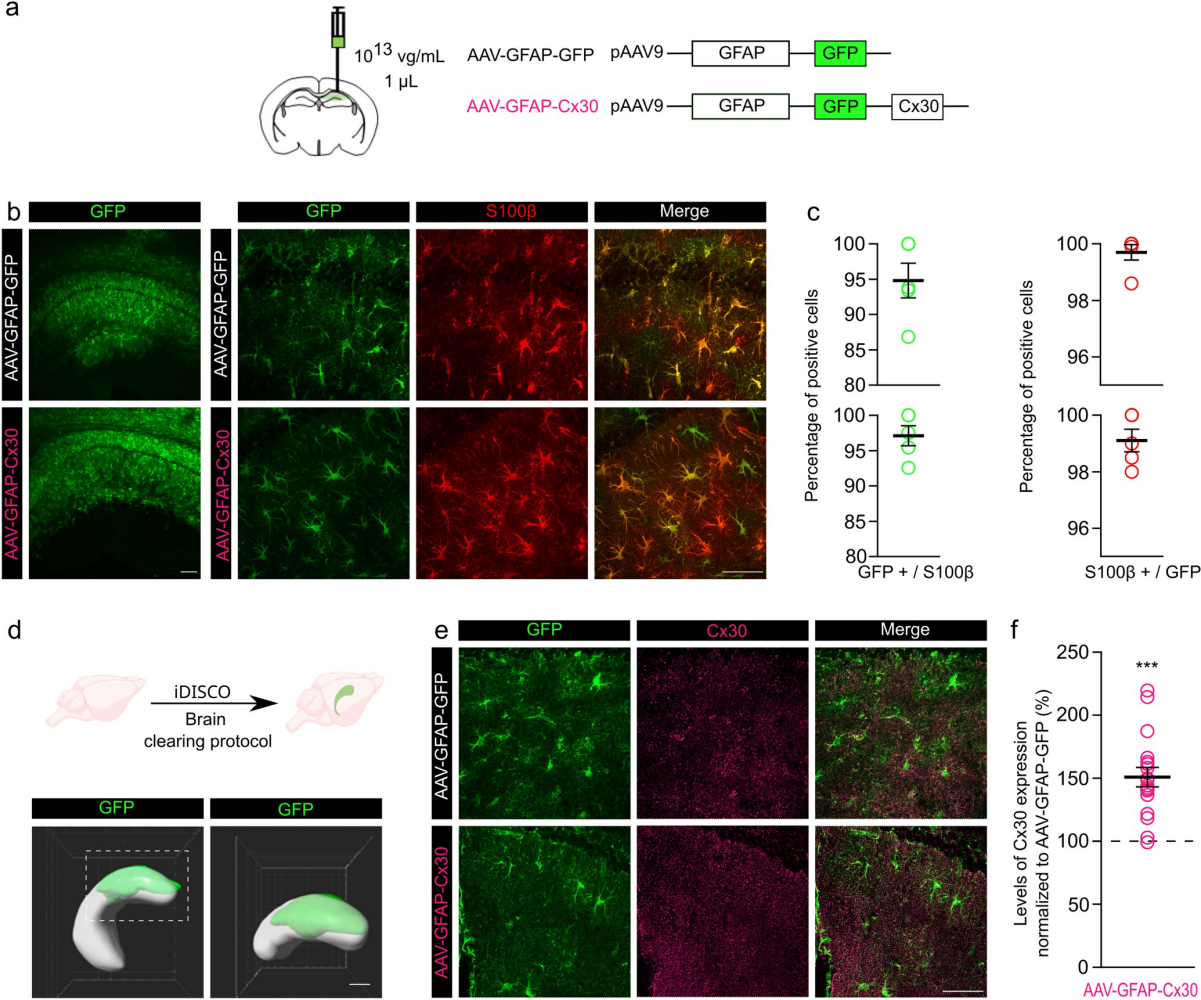
Increased expression of Cx30 in astrocytes from the dorsal hippocampus using in vivo local injection of AAV. **(a)** Schematic of the experimental procedure and of the viral vector constructs. (**b)** Confocal images of the CA1 region after injection of AAV-GFAP-GFP or AAV-GFAP-Cx30 and immunolabeling of GFP (green) at low magnification (left panel), scale bar: 20 μm or high magnification (right panels), scale bar: 50 μm. Note that the immunolabeling of S100β (red) strongly overlaps GFP expression. (**c)** Quantification of GFP+/S100β (*n =* 5 ROI) and S100β+/GFP (*n* = 5 ROI) positive cells after injection of AAV-GFAP-GFP (top) or AAV-GFAP-Cx30 transduction (bottom). **(d)** Reconstructed volume in the hippocampus of the viral transduction (green) after brain clearing protocol iDISCO (left) and at higher magnification of its dorsal part (right). Scale bar: 500 μm**. (e)** Confocal images of the CA1 region after injection of AAV-GFAP-GFP or AAV-GFAP-Cx30, and immunolabeling of GFP (green) and Cx30 (purple), scale bar: 50 μm (left panels). (**f)** Quantification of Cx30 expression after AAV-GFAP-Cx30 transduction normalized to AAV-GFAP-GFP (*n =* 30 ROI). Individual numerical values are indicated in S1 Data. ***p < 0.001, Welch *t* test.

Effects of increased Cx30 expression in astrocytes were investigated following unilateral injection of adeno-associated vectors (AAVs) into the right hippocampus. AAV-GFAP-Cx30 (Cx30-increased condition) or AAV-GFAP-GFP (control condition) constructs were expressed under the GFAP promoter to specifically transduce GFAP-expressing astrocytes (Fig 1A). To assess the efficiency of astrocyte transduction, we quantified the proportion of cells coexpressing GFP and S100β, a largely expressed astroglial marker, using immunostaining. We found that approximately 99% of GFP^+^ cells were S100β^+^ (Fig 1B and 1C). Reciprocally, we observed that approximately 96% of S100β^+^ cells were GFP^+^, indicating that the designed AAV selectively targeted astrocytes, and, virtually, all these cells were transduced. Histological controls performed 2 weeks after AAV injection confirmed that all injection sites were confined to the CA1 dorsal hippocampus. To evaluate the extent of the viral transduction, 3D reconstructions of transparent mouse brains were performed and revealed that approximately 30% of the hippocampus expressed the viral construct, specifically localized in its dorsal part (Fig 1D). Quantification of increased Cx30 expression by viral transduction was next performed by Cx30 immunolabeling (Fig 1E). Analysis of brain sections after AAV-GFAP-Cx30 transduction was performed and normalized to quantification following AAV-GFAP-GFP transduction. This revealed a 50% increase in Cx30 expression (Fig 1F), indicating an upregulation within a physiological range. The physiological amplitude of the variations in Cx30 levels was indeed previously determined in acute hippocampal slices, and a 50% decrease was found after blocking spontaneous action potential firing, whereas a 40% increase occurred after disinhibition, as assessed by western blotting and immunohistochemistry [18]. In addition, mice exposed to an enriched environment show enhanced Cx30 expression [19].Thus, an increased level of Cx30 after AAV-GFAP-Cx30 transduction corresponds to a variation within a physiological range.

### Cx30 regulates astrocytic currents and the size of the astroglial network

**Fig 2:**
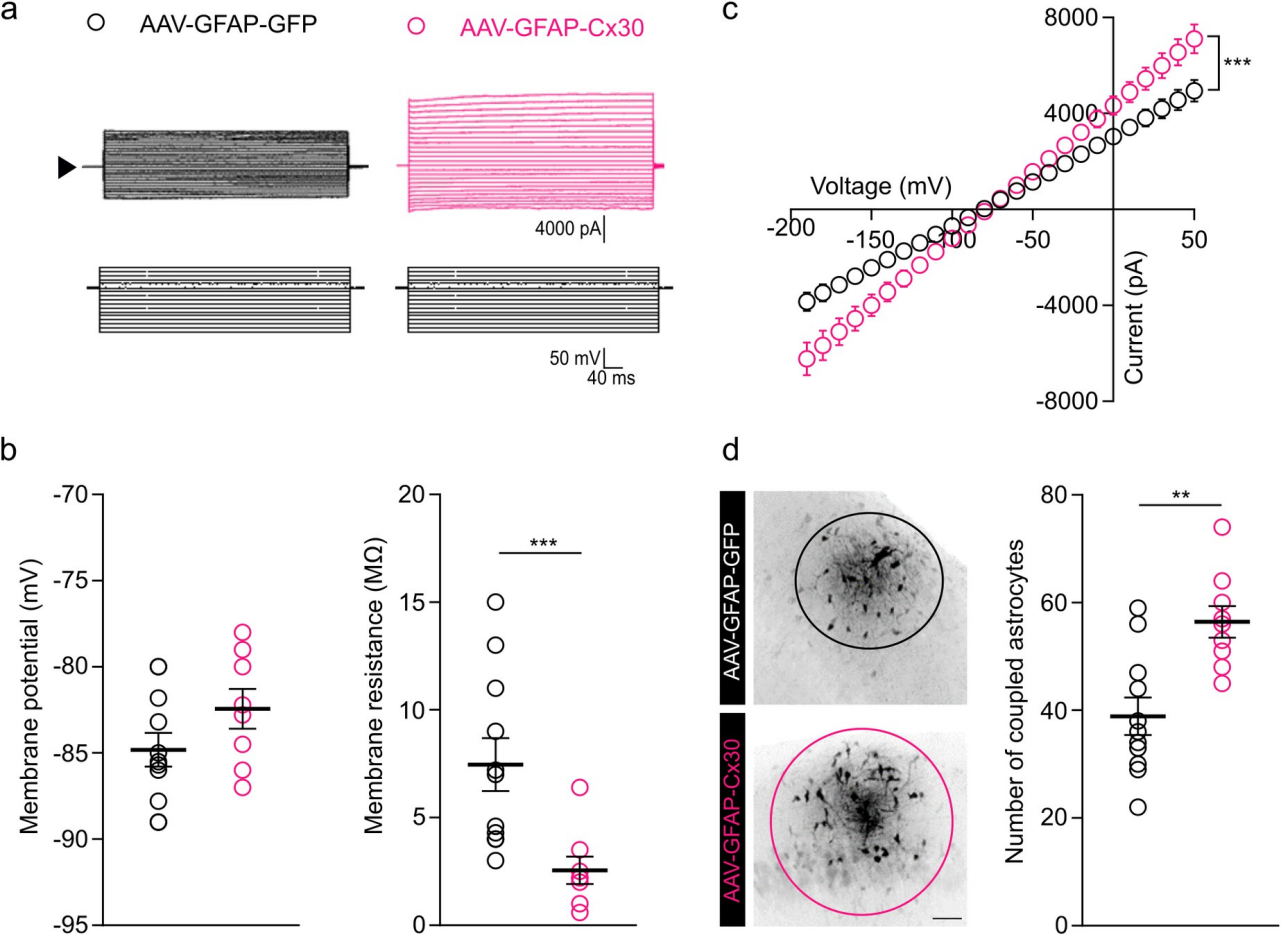
Upregulation of Cx30 increased passive astrocytic currents and the size of the astroglial network. **(a)** Representative whole-cell current profiles evoked by current injections from GFP-labeled astrocytes clamped at −80 mV. (**b**) The membrane potential of astrocytes is unchanged after AAV-GFAP-Cx30 (*n =* 9 astrocytes from 6 mice) compared to AAV-GFAP-GFP (*n =* 11 astrocytes from 5 mice) injection (*p* = 0.1572, Mann–Whitney test). Membrane resistance is decreased after AAV-GFAP-Cx30 (*n* = 9 astrocytes from 6 mice) compared to AAV-GFAP-GFP (*n* = 11 astrocytes from 5 mice) injection (***p < 0.001, Mann–Whitney test). **(c**) Current–voltage curve (IV curve) of astrocytes. Astrocytic currents were increased after AAV-GFAP-Cx30 (*n* = 9 astrocytes from 6 mice, pink) compared to AAV-GFAP-GFP transduction (*n* = 11 astrocytes from 5 mice, black) injection (***p < 0.001, interaction: F (24,425) = 9.0, two-way ANOVA). (**d)** Images of gap junction–mediated biocytin coupling in CA1 from brain slices following injection of AAV-GFAP-GFP (black) or AAV-GFAP-Cx30 (pink). Scale bar: 50 μm. Gap junction coupling is significantly enhanced, as quantified by counting the number of coupled cells after 10 min of biocytin injection in a single astrocyte through a patch pipette after AAV-GFAP-Cx30 (*n =* 9 slices from 6 mice) transduction compared to AAV-GFAP-GFP transduction (*n* = 11 slices from 5 mice). Individual numerical values are indicated in S1 Data. **p = 0.0012, Welch *t* test.

To study the impact of increased Cx30 expression on the functional properties of astrocytes, we recorded their electrophysiological properties (Fig 2A). We first characterized the membrane properties and found that Cx30 upregulation in astrocytes did not affect their membrane potential but decreased their membrane resistance (approximately 65%) (Fig 2B) and whole-cell passive currents (linear current–voltage relationship; Fig 2C). As astroglial Cx30 is one of the two gap junction subunits contributing to the direct intercellular coupling of astrocytes, we next investigated whether the increased Cx30 expression alters the size of the astroglial network. To do so, we infused biocytin, a low molecular weight tracer permeable to gap junction channels, for 10 min into single astrocytes via a patch pipette and found that the Cx30 upregulation markedly enhanced astroglial coupling, as assessed by the significantly increased number of coupled astrocytes (approximately 30%; Fig 2D).

### Upregulation of astroglial Cx30 decreases hippocampal excitatory synaptic activity

**Fig 3:**
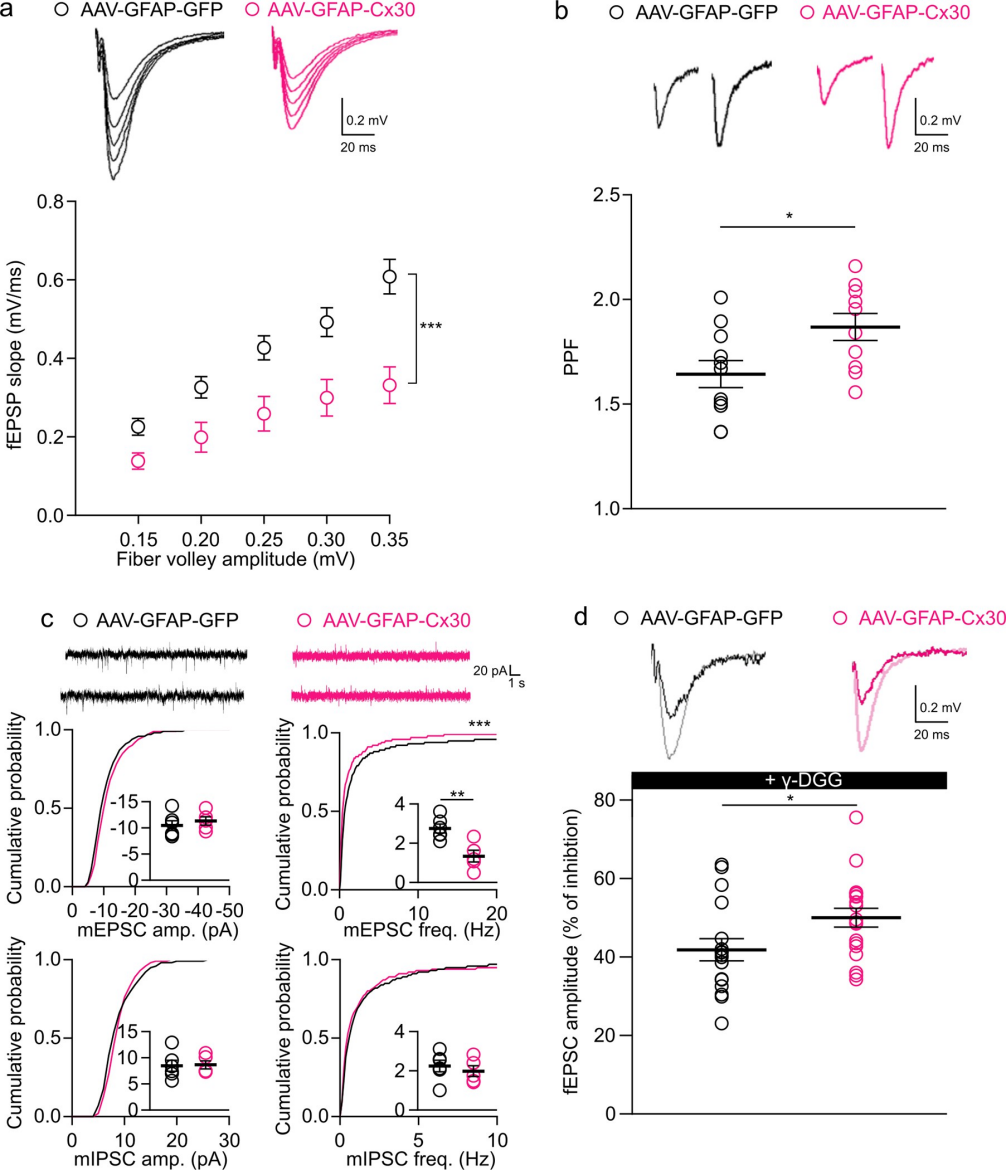
Cx30 upregulation in astrocytes alters excitatory synaptic transmission at CA1 Schaffer collateral synapses. **(a)** Input–output curves for basal synaptic transmission are illustrated in the sample traces and the graph below. fEPSP slope was decreased for AAV-GFAP-Cx30 (*n =* 8 slices from 5 mice) compared to AAV-GFAP-GFP (*n =* 7 slices from 5 mice; AAV: ***p < 0.001, F (1, 65) = 31,8; fiber volley: ***p < 0.001, F (4, 65) = 17,4, two-way ANOVA). (**b)** PPF ratio (2 stimulations, interval 40 ms) and representative traces. PPF was increased for AAV-GFAP-Cx30 (*n =* 11 slices from 5 mice) compared to AAV-GFAP-GFP (*n =* 10 slices from 5 mice). *p* < 0.05, unpaired *t* test. (**c)** (Upper) Sample traces from whole pyramidal cell recording. (Lower) Cumulative probability and mean values of mEPSCs and mIPSCs amplitudes and frequencies in both mice with AAV-GFAP-GFP (*n* = 6 from 5 mice) or AAV-GFAP-Cx30 (*n* = 5 from 5 mice). *p < 0.01, Mann–Whitney test, ***p < 0.001 K = 0.5466, Kolmogorov–Smirnov test. (**d)** Synaptic glutamate levels revealed by γ-DGG (0.5 mM) inhibition of EPSC amplitude were reduced in mice with AAV-GFAP-Cx30 mice (*n* = 19 slices from 6 mice) compared to mice transduced with AAV-GFAP-GFP (*n* = 17 slices from 5 mice). Individual numerical values are indicated in S1 Data. *p < 0.05; Mann–Whitney test. AAV, adeno-associated vector; EPSC, excitatory postsynaptic current; fEPSP, field excitatory postsynaptic potential; mEPSC, miniature excitatory postsynaptic current; mIPSC, miniature inhibitory postsynaptic current; PPF, paired pulse facilitation; γ-DGG, γ-D-glutamylglycine.

As astrocytes are key regulatory elements of neuronal activity, we examine whether increased Cx30 expression regulates synaptic activity. We first measured basal evoked-synaptic responses at CA1 Schaffer collateral synapses. To do so, we compared the amplitude of the presynaptic fiber volley (input) to the slope of the field excitatory postsynaptic potential (fEPSP) in acute hippocampal slices from AAV-GFAP-Cx30 or AAV-GFAP-GFP injected mice. We found an approximately 35% reduction in synaptic transmission in mice with increased astroglial Cx30 expression (Fig 3A). What might cause impaired synaptic transmission in mice with upregulated astroglial Cx30? To test for glutamate impairment, we first analyzed paired pulse facilitation (PPF), a form of short-term synaptic plasticity sensitive to changes in release probability. Using extracellular recordings of fEPSPs, we found that PPF was increased by approximately 20% in mice with upregulated astroglial Cx30, indicating a decrease in the probability of presynaptic release (Fig 3B). To further assess alteration in transmitter release, we recorded miniature excitatory postsynaptic currents (mEPSCs) in CA1 pyramidal cells and found a strong reduction in their frequency (approximately 45%), with no change in their amplitude (Fig 3C), known to reflect vesicular glutamate content and/or the postsynaptic response. To directly test for changes in synaptic glutamate levels in mice with upregulated Cx30, we used γ-D-glutamylglycine (γ-DGG), a low-affinity and competitive antagonist of AMPARs, at nonsaturating concentration (500 μM), and whose potency depends on glutamate levels. γ-DGG inhibition of synaptically evoked EPSCs was higher in CA1 pyramidal cells from mice with upregulated Cx30 than from control mice (Fig 3D). These results indicate that upregulation of astroglial Cx30 selectively alters excitatory synaptic transmission. Altogether, these data suggest that the decreased excitatory synaptic transmission in mice with upregulated Cx30 results from reduced synaptic glutamate levels rather than from postsynaptic defects.

### Enhanced expression of astroglial Cx30 impairs long-term synaptic plasticity and recognition memory

**Fig 4:**
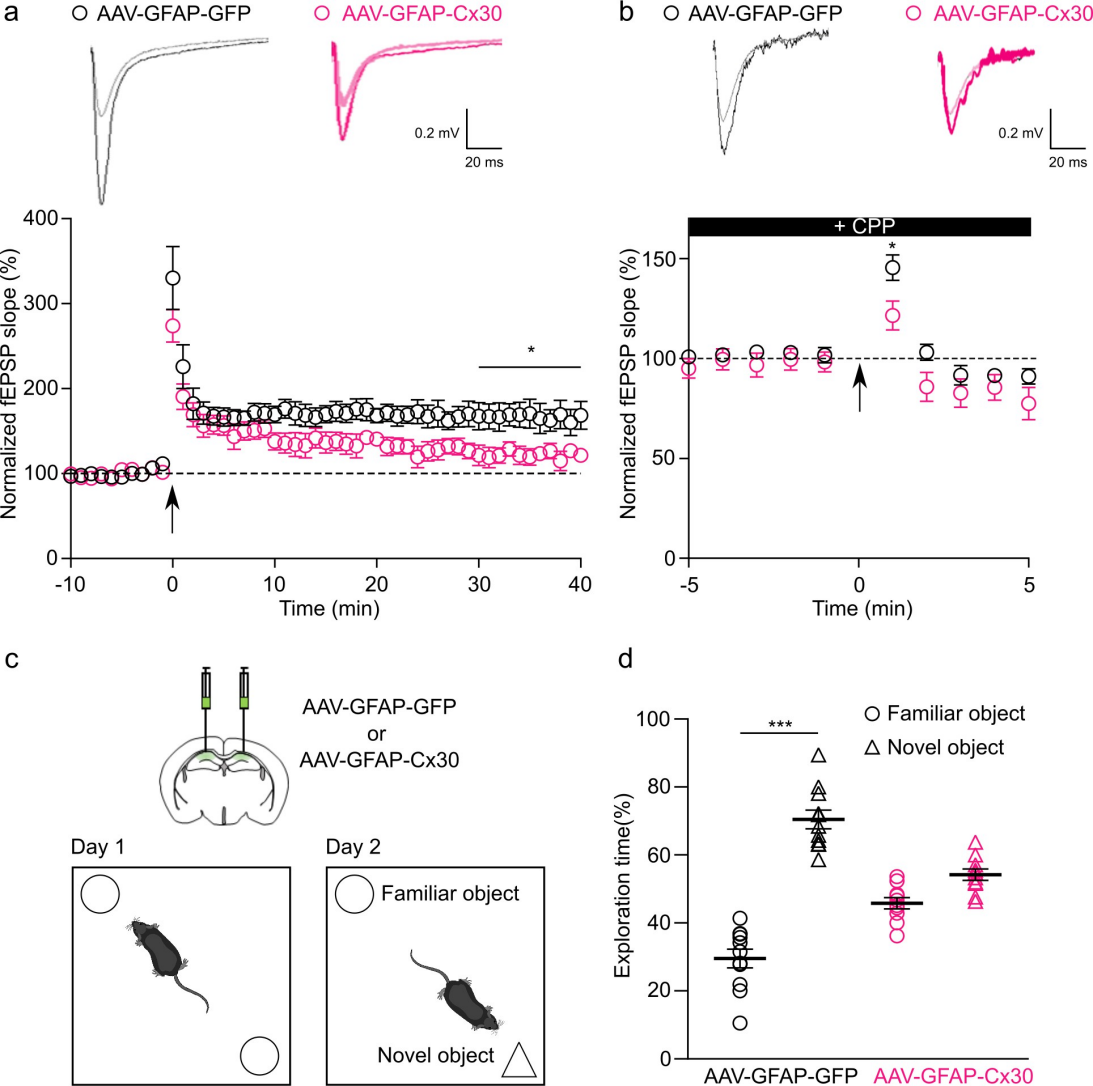
Upregulation of astroglial Cx30 decreased LTP and recognition memory. **(a)** Tetanus-induced LTP curves (arrow, two 100 Hz tetani for 1 s, interval 20 s) and representative traces. LTP maintenance for the last 10 min (30–40 min) was decreased for AAV-GFAP-Cx30 (*n =* 9 slices from 5 mice) compared to AAV-GFAP-GFP (*n* = 8 slices from 5 mice); *p < 0.05, Welch’s *t* test). **(b**) Tetanus-induced PTP curves (arrow, two 100 Hz tetani for 1 s, interval 20 s and representative traces under CPP (20 μM). PTP was decreased for AAV-GFAP-Cx30 (*n* = 12 slices from 5 mice) compared to AAV-GFAP-GFP (*n* = 12 slices from 5 mice; *p < 0.05, Welch’s *t* test). (**c)** Schematic of NOR memory test after bilateral hippocampal injections of AAV-GFAP-GFP or AAV-GFAP-Cx30 in mice. (**d)** Percentage exploration time for familiar versus novel object for AAV-GFAP-GFP (*n* = 11 mice) and AAV-GFAP-Cx30 (*n* = 10 mice). Individual numerical values are indicated in S1 Data. ***p < 0.001, Kruskal–Wallis multiple comparison test. CPP, 3-(RS)-(2-carboxypiperazin-4-yl)-propyl-1-phosphonic acid; LTP, long-term potentiation; NOR, novel object recognition; PTP, posttetanic potentiation.

Because Cx30 regulates synaptic efficacy, we then investigated its involvement in long-term synaptic plasticity. Following tetanic stimulation of Schaffer collaterals, we found that the amplitude of long-term potentiation (LTP) was significantly reduced (approximately 60%) in mice expressing increased levels of astroglial Cx30 (Fig 4A). To determine whether this alteration resulted from a defect in LTP induction, we analyzed posttetanic potentiation (PTP), a form of short-term plasticity resulting from an increase in neurotransmitter release induced by tetanic stimulation of Schaffer collaterals while blocking postsynaptic N-methyl-d-aspartate (NMDA) receptors (3-(RS)-(2-carboxypiperazin-4-yl)-propyl-1-phosphonic acid (CPP), 20 μM). We found that the amplitude of PTP was significantly decreased in mice with upregulated astroglial Cx30 (Fig 4B), indicating an impairment in LTP induction resulting from reduced neurotransmitter release evoked by the tetanus. Altogether, these results show that upregulating Cx30 in astrocytes reduces both excitatory synaptic transmission and long-term plasticity.

Hippocampal long-term synaptic plasticity underlies learning and memory, including recognition memory [22]. Because synaptic glutamate is important for recognition memory [21], we investigated in vivo the contribution of an enhanced expression of astroglial Cx30 in this form of memory. To do so, we subjected adult mice bilaterally injected with AAV-GFAP-Cx30 or AAV-GFAP-GFP to a novel object recognition (NOR) test (Fig 4C). Because mice have an innate preference for novelty, we assessed recognition memory by quantifying the relative time spent exploring a novel versus a familiar object. Whereas control AAV-GFAP-GFP-injected mice spent more time exploring the novel object, the preference of the novel over the familiar object was absent in AAV-GFAP-Cx30-injected mice, pointing to an altered recognition memory (Fig 4D).

### Astroglial Cx30 enhanced expression impairs CA1 pyramidal cells excitability and action potential properties

**Fig 5:**
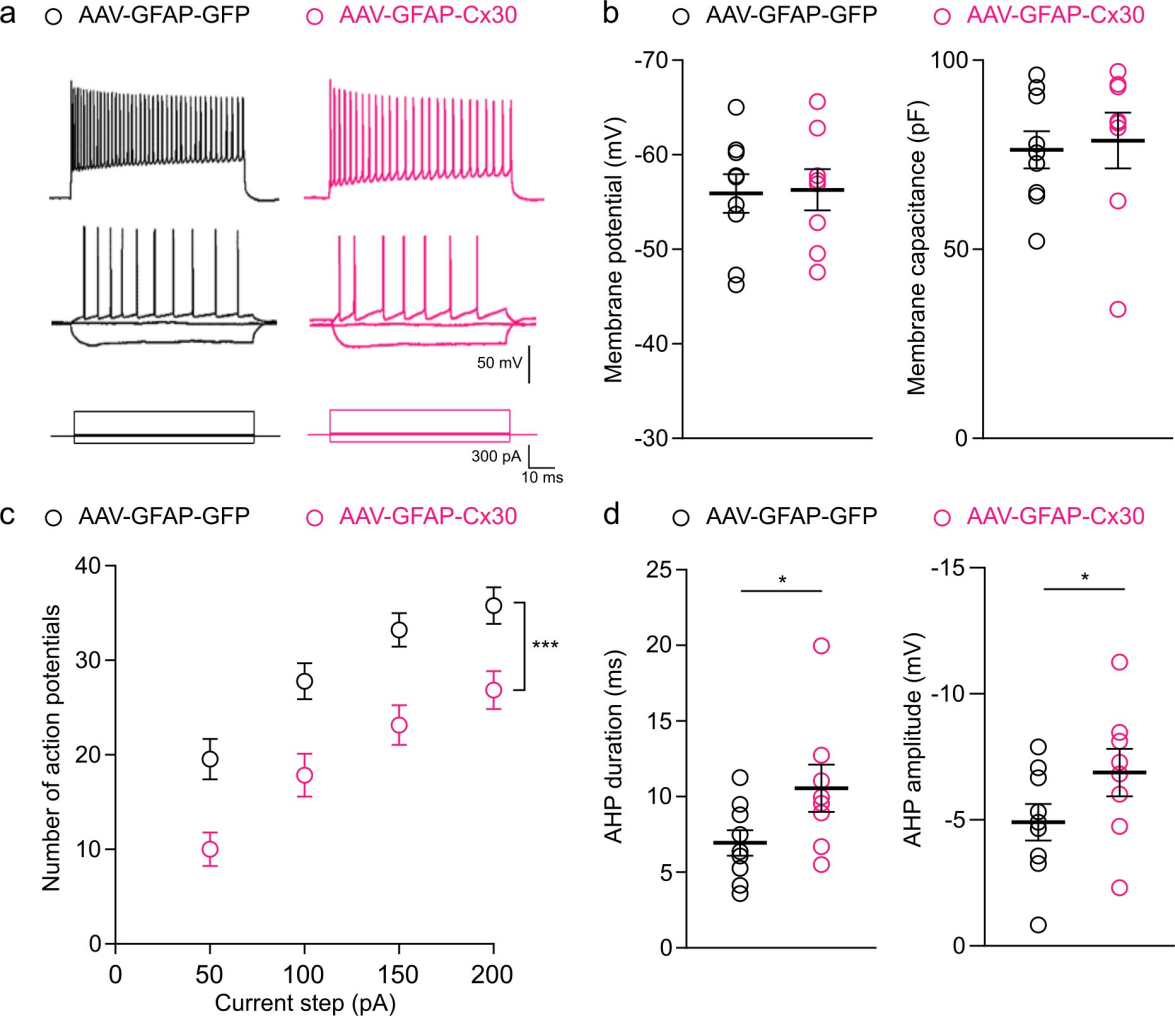
Upregulation of Cx30 impairs pyramidal cell excitability and action potential properties. **(a)** Typical electrophysiological response of CA1 pyramidal cells from control and Cx30 enhanced expression mice. CA1 pyramidal cells were recorded using whole-cell patch-clamp recordings in response to current pulses (bottom traces). Strong depolarizing current (top trace) evoked a high and sustained firing. (**b)** Resting membrane potential and membrane capacitance were unchanged in CA1 pyramidal cells from mice injected with AAV-GFAP-GFP (*n =* 9 neurons from 5 mice) or AAV-GFAP-Cx30 (*n* = 8 neurons from 5 mice; *p* = 0.9825 for membrane potential, *p* = 0.4807 for membrane capacitance, Mann–Whitney test). (**c)** Excitability curve with the number of action potentials evoked by current step for AAV-GFAP-GFP (*n* = 9 neurons from 5 mice) and AAV-GFAP-Cx30 (*n* = 8 neurons from 5 mice) transduced mice (AAV: ***p < 0.0001, F (1, 71) = 46,6; current step: ***p < 0.0001, F (4, 71) = 101.8, two-way ANOVA). (**d)** AHP duration and amplitude were increased for AAV-GFAP-Cx30 (*n* = 8 neurons from 5 mice) compared to AAV-GFAP-GFP (*n* = 9 neurons from 5 mice) transduced mice. Individual numerical values are indicated in S1 Data. *p < 0.05, Mann–Whitney test. AAV, adeno-associated vector; AHP, afterhyperpolarization; fEPSP, field excitatory postsynaptic potential.

We then investigated how does increased expression of astroglial Cx30 alters synaptic glutamate levels. To this end, we tested for changes in CA1 pyramidal cell intrinsic membrane properties and excitability. Recordings of pyramidal cell electrophysiological responses to hyperpolarizing and depolarizing current pulses and analysis of 28 discriminative electrophysiological parameters did not reveal changes in their passive membrane properties (resting membrane potential and capacitance) in mice with enhanced expression of astroglial Cx30 (Fig 5A and 5B). However, the number of action potentials evoked by depolarizing pulses was lower in CA1 pyramidal cells from AAV-GFAP-Cx30-injected mice (Fig 5C). Previous work on mice deficient for astroglial connexins reported a decrease in the amplitude and duration of CA1 pyramidal cells afterhyperpolarization (AHP) [23–25], i.e., the refractory period following action potential discharges during which neuron membrane potential is hyperpolarized (undershoot). We thus investigated AHP in mice with upregulated Cx30 and found an increased amplitude (approximately 40%) and duration (approximately 15%). These results indicate that upregulation of astroglial Cx30 alters pyramidal cell excitability and action potential properties.

### Modulation of AHP in pyramidal cells restores normal excitatory synaptic transmission in mice with upregulated astroglial Cx30

AHP inhibits the rapid initiation of subsequent action potentials and thereby regulates neuronal excitability by setting the frequency of action potential discharge [26,27]. AHP has several components and is mediated by voltage-gated K^+^ channels, such as Kv7.2/7.3/KCNQ subunits or HCN channels [26]. Kv7.2/7.3/KCNQ channels are abundantly expressed in CA1 and CA3 pyramidal cells and generate the M-current (I(M)) underlying the AHP medium component, whereas HCN channels mediate the voltage sag following hyperpolarization [7,18]. In addition, here, we did not observe any significant effect on the sag response of pyramidal cells from mice with upregulated Cx30 compared to pyramidal cell sag responses from control mice (88.82 ± 2.21 versus 83.72 ± 2.86; *p* = 0.16), while it has recently been reported that astroglial gap junctions strengthen hippocampal network activity by sustaining AHP via Kv7.2/7.3/KCNQ channels [25].

**Fig 6:**
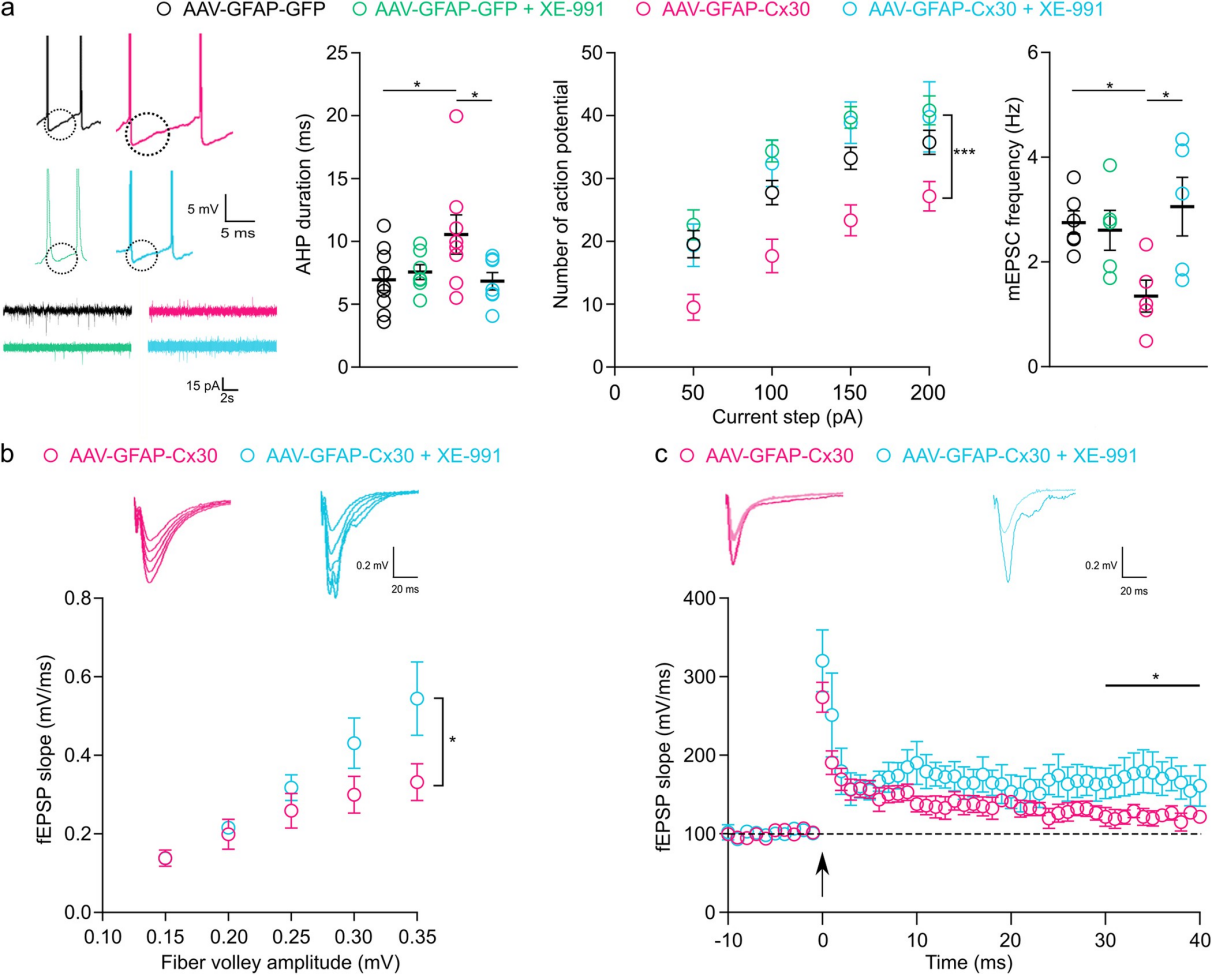
Reducing AHP duration in mice with upregulated astroglial Cx30 restores pyramidal cell excitability and excitatory synaptic transmission. **(a)** (Left) Sample traces of whole CA1 pyramidal cell recording from mice stereotaxically injected with AAV in the hippocampus in the presence or not of XE-991. The dotted circles delineate the AHP. (Middle Left) AHP durations in CA1 pyramidal cells was unchanged after the application of XE-991 in mice injected with AAV-GFAP-GFP (*n =* 8 neurons from 5 mice), AAV-GFAP-GFP with XE-991 (10 μM, *n =* 7 neurons from 5 mice), and AHP was restored by the application of XE-911 in AAV-GFAP-Cx30 mice (*n =* 8 neurons from 5 mice), AAV-GFAP-Cx30 with XE-991 (10 μM, *n* = 7 neurons from 5 mice). *p < 0.05, Mann–Whitney test. (Middle right) Excitability curve with the number of action potentials evoked by current step for mice injected in the hippocampus with AAV-GFAP-GFP (*n* = 9 neurons from 5 mice), AAV-GFAP-Cx30 (*n* = 8 neurons from 5 mice), or AAV-GFAP-Cx30 + XE-991 (*n =* 8 neurons from 5 mice) (AAV: ***p < 0.001, F (2, 93) = 18.6; current step: ***p < 0.001, F (4, 93) = 96.38, two-way ANOVA). (Right) mEPSCs frequencies were not affected by the application of XE-911 in mice injected with AAV-GFAP-GFP (*n =* 6 from 5 mice) AAV-GFAP-GFP with XE-991 (10 μM, *n* = 5 neurons from 5 mice), but mEPSC frequencies were restored by the application of XE-991 in mice injected with AAV-GFAP-Cx30 (*n* = 5 from 5 mice) AAV-GFAP-Cx30 with XE-991 (10 μM, *n* = 5 neurons from 5 mice). *p < 0.05, Mann–Whitney test. (**b**) Input–output curves for basal synaptic transmission. fEPSP slope was increased after the application of XE-991 in mice injected in the hippocampus with AAV-GFAP-Cx30 (*n* = 10 slices recording from 5 mice) compared to mice injected with AAV-GFAP-Cx30 (*n* = 8 slices recording from 5 mice) (XE-991: *p < 0.05, F (1, 75) = 6.623; fiber volley: ***p < 0.001, F (4, 75) = 10.85, two-way ANOVA). (**c**) Tetanus-induced LTP curves (arrow, two 100 Hz tetani for 1 s, interval 20 s) and representative traces. LTP maintenance for the last 10 min (30–40 min) was restored for AAV-GFAP-Cx30 in the presence of XE-991 (*n* = 9 slices from 5 mice) compared to AAV-GFAP-Cx30 (*n* = 7 slices from 5 mice). Individual numerical values are indicated in S1 Data. *p < 0.05, Welch’s *t* test. AAV, adeno-associated vector; AHP, afterhyperpolarization; fEPSP, field excitatory postsynaptic potential; LTP, long-term potentiation; mEPSC, miniature excitatory postsynaptic current.

We thus investigated whether the decreased neuronal excitability in mice with enhanced expression of astroglial Cx30 resulted from increase in medium AHP component. To do so, we tested whether a reduction of AHP by XE-991 (10 μM), a specific Kv7.2/7.3/KCNQ K^+^ channel inhibitor altering AHP medium component [26–28], could rescue normal excitability of CA1 pyramidal cells in mice with increased levels of astroglial Cx30. To this end, we first tested whether XE-991 alters CA1 pyramidal cell properties and synaptic transmission in control mice transduced with AAV-GFAP-GFP. We found that XE991 had no effect on CA1 pyramidal cell resting potential, membrane resistance, and AHP duration in these mice (Fig 6A). Furthermore, XE-991 also had no effect on cell excitability and basal synaptic transmission, assessed by the number of action potential per current step intensity and mEPSC frequency (Fig 6A), which were both altered by Cx30 upregulation, as mentioned above (Figs 3C and 5C). Altogether, these data indicate that in control condition, XE-991 had no effect on CA1 pyramidal cell properties (Fig 6A).

We then determined the effects of XE-991 on the properties of CA1 pyramidal cells from mice with upregulated Cx30 expression, transduced with AAV-GFAP-Cx30. We found that XE-991 decreased AHP duration (approximately 35%) to control values and that this was sufficient to restore normal excitability and mEPSCs frequency in CA1 pyramidal cells (Fig 6A).

Subsequently, we tested whether the alteration in AHP mediating the decreased neuronal excitability accounts for the reduced excitatory synaptic transmission in mice with upregulated astroglial Cx30. To this end, we recorded fEPSPs evoked by Schaffer collateral stimulation in the presence of XE-991 and found that this also rescued excitatory synaptic transmission to control levels in AAV-GFAP-GFP-injected mice (Fig 6B). This rescue is observed for high amplitudes of fiber volley when differences of fEPSP slope are more pronounced between control and enhanced Cx30 expression conditions (Fig 3A). Moreover, we found in mice with upregulated Cx30 that the AHP reduction induced by XE-991 fully rescued LTP (Fig 6B). These results indicate that Cx30 upregulation in astrocytes decreases glutamatergic synaptic transmission via AHP regulation of neuronal excitability.

## Discussion

By combining local molecular approaches in the CA1 region of the hippocampus and electrophysiological recordings, we here show that increased expression, in a physiological range, of Cx30 selectively in astrocytes decreases excitatory synaptic transmission and alters LTP induction, as well as recognition memory. To some extent, these results are similar to those obtained in mice deficient for astroglial Cx30, where hippocampal excitatory synaptic strength was also reduced, as well as long-term synaptic plasticity and memory [21].

### Levels of Cx30 regulate synaptic glutamate concentration, hippocampal excitatory synaptic strength, plasticity, and memory

Here, we found that enhanced expression of Cx30 in astrocytes decreases the levels of glutamate released at synapses, leading to impaired excitatory synaptic transmission and LTP induction and translating to the behavioral level in a loss of recognition memory.

This reduced extracellular glutamate is associated with an increased size of the astroglial network that allows for better diffusion of glutamate and thus increased uptake. This suggests that the release probability could be, at least partially, regulated by the size of the gap junction–mediated astroglial network. The extent of these networks indeed determines the diffusion of the cleared neuroactive molecules, i.e., K^+^ and glutamate, in these networks, and, thus, the extracellular levels of these molecules, which can regulate neuronal excitability and release probability. This hypothesis is based on previous work showing that gap junctional networking facilitates extracellular glutamate and K^+^ removal during synaptic activity through modulation of astroglial clearance rate [2]. Disconnected astrocytes in mice deficient for both astroglial Cxs indeed fail to properly remove the enhanced extracellular glutamate and K^+^ levels during synaptic activity, as indicated by the slower decay kinetics of both, glutamate transporter currents and membrane potential depolarization, reflecting K^+^ uptake, evoked synaptically in astrocytes. Thus, although disconnected astrocytes can still take up released K^+^ and glutamate during synaptic activity, they are unable to redistribute them via the network due to the absence of gap junction channels. This alters the concentration gradients for K^+^ and glutamate and, thus, their clearance efficiency.

Interestingly, a similar decrease in synaptic glutamate levels was observed in constitutive Cx30-deficient mice, however, via a different mechanism [21]. In these mice, release probability was indeed unaltered, but astroglial glutamate transport was strongly increased. This directly reduced synaptic glutamate levels and, thus, excitatory synaptic transmission and induction of LTP, which translated into a loss of contextual fear memory. Remarkably, the regulation of glutamate transport by Cx30 was independent of its channel function and was mediated by structural changes, which controlled the synaptic insertion of fine astroglial processes, known to be enriched in glutamate transporters.

Thus, although Cx30 upregulation or deficiency apparently leads to similar phenotypes at the synaptic and behavioral levels, namely reduced synaptic glutamate levels and excitatory synaptic transmission associated with defective LTP induction and memory (recognition and contextual fear memory, respectively), the underlying molecular mechanisms likely differ. This suggests that Cx30, whose expression can be dynamically up- or downregulated in various physiological or pathological contexts, is a critical regulator of synaptic function and behavior, with an optimal expression level required for proper synaptic and cognitive functions.

In future studies, it will be interesting to quantify the impact of the size of the astrocyte network on extracellular levels of K+ using K-sensitive electrodes in control and enhanced Cx30 expression conditions. Quantifying K+ and glutamate uptake would also confirm that the increased size of the astrocytic network acts directly on uptake facilitation. It would also be interesting to study to what extent the activation of metabotropic glutamate receptors (mGluRs) in pyramidal cells participates in the inhibition of Kv channels. Finally, testing whether increased Cx30 levels have a role in the structural properties of astrocytes and their coverage of synapses using super-resolution STED microscopy would be important to assess the involvement of channel and nonchannel function of Cx30 in the regulation of neuronal activity and plasticity that we here describe. Altogether, this would reinforce the possible mechanism linking the reduced neuronal excitability to the increased size of the astrocytic network via an enhanced K+ and glutamate uptake.

### Enhanced Cx30 expression in astrocytes regulates action potential properties and neuronal excitability

To evaluate the impact of astroglial Cx30 upregulation on neuronal properties, we recorded the electrophysiological properties of neighboring pyramidal cells. We found that Cx30 upregulation in astrocytes reduces the frequency of action potential discharge in CA1 pyramidal cells via modulation of the medium AHP, as shown by the effect of XE-991, an inhibitor of Kv7.2/7.3/KCNQ channels mediating the medium AHP, which fully rescued excitability in CA1 pyramidal cells from mice with upregulated Cx30 expression. Interestingly, astroglial gap junctions mediated by Cx30 and Cx43 were also recently reported to regulate neuronal activity, i.e., strengthen hippocampal network activity, by controlling AHP via KCNQ channels [25].

Remarkably, we here report that XE-991 also restored excitatory synaptic transmission in mice with upregulated Cx30. These results are consistent with previous observations indicating that modulation of the K^+^ voltage-dependent channels K_V_7.2 and K_V_7.3 can regulate neuronal excitability [26,29–31]. Kv7 channels are voltage-sensing channels; they open and close upon changes in transmembrane potential to selectively let K^+^ ions pass through the channel. Hence, an increase in extracellular K^+^ favors the closure of these channels, thereby enhancing neuronal activity [32]. In the pancreas, gap junction channels formed by the Cx36 subunit were shown to regulate K^+^ channels to coordinate insulin secretion [33]. Notably, K_V_7 channels are also negatively regulated by activation of group I mGluRs [34]. Interestingly, activation of mGluRs suppresses the AHP and increases the magnitude of LTP [35,36]. Hence, it is possible that enhanced glutamate and possibly K^+^ uptake by a larger gap junction–mediated astroglial network in mice with increased expression of Cx30 may inhibit the negative regulations of K_V_7 channels by activation of mGluRs and reduction of extracellular K^+^ levels. The decrease in K_V_7 channel’s closure might thereby increase K^+^ efflux and thus reduce neuronal excitability and synaptic transmission.

### Putative physiological role of increasing astrocytic networks to limit neuronal activity

Physiologically, it has already been shown that hippocampal Cx30 protein levels, as well as astrocytic network size, can be modulated by neuronal activity [18,37], suggesting that there is a close relationship between astrocytic network size and the activation of underlying neuronal networks. However, this modulation is complex, as it differentially impacts the principal cells and the interneurons [23]. In addition, Cx30 can also act via other mechanisms, such as signaling and protein interactions. It has indeed recently been shown that the increase in Cx30 levels that occurs between P10 to P50 controls the closure of critical period in the mouse visual cortex, via a signaling pathway regulating the extracellular matrix and interneuron maturation [38].

Altogether, our results reveal that while neuronal activity increases the functional connectivity of the gap junction–mediated astroglial network by increasing the permeability of gap junction channels [37,39], directly increasing the number of these channels via increased expression of astroglial Cx30 on the contrary reduces neuronal excitability and synaptic transmission via modulation of K_V_7 channel activity. These results highlight the existence of a negative retro-control loop to maintain neuronal excitability within physiological ranges. We thus propose that the size of the astroglial network has a physiologically optimized configuration to tightly and appropriately regulate neuronal functions and networks. They are finely regulated, neither too large nor too reduced, to respond to neuronal demand, and allow brain efficient neuronal network activities and processes such as learning and memory.

## Methods

### Mice

Male and female C57BL/6J mice at P26 to 32 were used for electrophysiological experiments and between 8 and 10 weeks for NOR experiments. The effects were similar in both mice groups; thus, data obtained from male and female mice were pooled. All applicable international, national, and/or institutional guidelines for the care and use of animals were followed. Adult mice were housed under a 12-h light/12-h dark circle, with ad libitum access to food and water, in accordance with European Community (Directive 2010-63/EEC) and French regulations (Code Rural R214/87–130). Experimental procedures were approved by the local ethics committee and registered with the French Research Ministry (CEEA, Paris 6, #14604). A total of 102 mice were used for all experiments.

### AAV production and injections

The AAV-GFAP-Cx30 construct consisted in a transgene composed, from 5′ to 3′, of GFP, the 2A peptide coding sequence and mouse Cx30 cDNAs in a single open reading frame, was placed under the control of a GFAP-specific promoter in an AAV shuttle plasmid containing the inverted terminal repeats (ITRs) of AAV2 [14]. The 2A signal allowed that GFP and Cx30 were expressed as distinct polypeptides, thus preventing bias on Cx30 function and localization potentially induced by a fusion protein. The AAV-GFAP-GFP control vector encoded GFP only. Serotype 9 adeno-associated viruses (AAVs) particles were produced by transient cotransfection of HEK-293T cells, as previously described [40]. Viral titers were determined by quantitative PCR amplification of the ITR on DNase-resistant particles and expressed as vector genomes per ml (vg/ml). Juvenile mice were injected with AAV at P18 ± 2 days for 2 main reasons: At this time point, electrophysiological recordings are facilitated, and Cx30 has not yet reached its maxima expression level. Animals were deeply anesthetized with a mixture of ketamine (95 mg/kg, Merial) and xylazine (10 mg/kg, Bayer) in 0.9% NaCl and placed in a stereotaxic frame, with constant body heat regulation. Injections were unilaterally performed in the right hippocampus at the following stereotaxic coordinates: AP: −1.85 mm; L: 1.6 mm to the bregma at a depth of −1.5 mm to the skull. We injected 1 μL of virus AAV-GFAP-GFP or AAV-GFAP-Cx30 at 1.10^13^ vg/mL, with a 29-gauge blunt-tipped needle linked to a 2-μL Hamilton syringe by a polyethylene catheter, at a rate of 0.1 μL/min, with an automatic pump. After the injection, the needle was left in place for 5 min before being slowly removed. The skin was glued, and mice recovery was checked for the next 24 h.

### Electrophysiology

Two weeks after AAV injection, mice were decapitated. Their brains were quickly extracted and submerged in cold slicing artificial cerebrospinal fluid (aCSF, 4°C) containing (in mM): 119 NaCl; 2.5 KCl; 2.5 CaCl_2_; 26.2 NaHCO_3_; 1 NaH_2_PO_4_; 1.3 MgSO_4_; 11 D-glucose (pH 7.35). Brains were constantly oxygenated with 95% O_2_−5% CO_2_. Hippocampal brain slices (400 μm thick) were cut with a vibratome (VT1200S; Leica) and transferred to a constantly oxygenated (95% O_2_−5% CO_2_) holding chamber containing aCSF for at least 1 h prior to recording. Subsequently, individual slices were placed in a submerged recording chamber maintained at 32°C and perfused with oxygenated aCSF, and placed under an upright microscope (AxioScop, Zeiss) equipped with a Neo sCMOS camera (ANDOR technology) for observation. Only GFP fluorescent slices were recorded.

Extracellular fields and whole-cell patch-clamp recordings were performed. For extracellular field recordings, experiments were performed in the presence of picrotoxin (100 μM, Sigma), and a cut was made between CA1 and CA3 to prevent the propagation of epileptiform activity. Evoked postsynaptic potentials were induced by stimulating Schaffer collaterals (0.1 Hz, 0.1 s pulse duration), in CA1 stratum radiatum with ACSF-filled glass pipettes. Similar stimulation intensities were used to evoke fEPSPs in mice with normal or upregulated Cx30 levels. PPF was evoked by 2 repetitive stimulations at 40 ms interstimulus interval. LTP was induced by tetanic stimulation of Schaffer collaterals (2 trains of 100 Hz for 1 s, 20 s apart). PTP was induced with the same tetanic stimulation, but in presence of CPP (20 μM, Sigma).

Recordings in the whole-cell patch-clamp configuration were performed with patch-clamp pipettes (3 to 6 MΩ) filled with an internal solution containing (in mM): 129 K-gluconate; 10 EGTA; 10 HEPES and 2 ATP-Mg (pH 7.2 and 285 to 295 mOsm). About 2 mg/mL biocytin (Sigma) was added to this internal solution to assess the extent of gap junction–mediated astrocytic network. The recorded astrocytes were located in CA1 stratum radiatum. For each neuron, 28 electrophysiological parameters were measured according to the Petilla terminology [41]. For parameter 1 (**p1**), the resting membrane potential was measured immediately after passing to the whole-cell configuration. (**p2**) Input resistance (Rm) and (**p3**) the membrane time constant (τm) was determined at the beginning of the voltage response of a hyperpolarization current step (−10 pA, 800 ms). The time constant was determined by fitting this voltage response to a single exponential. (**p4**) The membrane capacitance (Cm) was calculated according to the equation Cm = τm / Rm. Under our conditions, injection of hyperpolarizing current pulses often induced a hyperpolarization-activated cationic current (Ih) that followed the initial hyperpolarization peak, known as a sag. (**p5**) R_hyp_ was measured as the slope of the linear portion of an I-V plot, measured at the beginning (0 to 0.1 s; −100 to 0 pA). (**p6**) R_sag_ was measured as the slope of the linear portion of a current–voltage (IV) plot, measured at the end of the hyperpolarizing current pulses (0.7 to 0.8 s; −100 to 0 pA). (**p7**) ΔR_Sag_ corresponds to (R_sag_ − R_hyp_) / R_sag_. (**p8**) Action potential threshold corresponded to the voltage threshold of the first action potential elicited by a current ramp. (**p9**) The rheobase was the minimum current that elicited an action potential. (**p10**) The first spike latency was computed as the time needed to elicit a spike after the onset of a current pulse corresponding to the rheobase. Neurons have been described to exhibit a wide range of firing behaviors around the threshold, some exhibiting bursting, adapting, regular, or irregular trains of action potentials. To describe this variety of behaviors with quantitative parameters, the interspaced intervals measured in response to the minimal current injection eliciting more than 3 action potentials were plotted and fitted to a linear curve. (**p11**) Adaptation (m_threshold_) was computed as the slope of the linear fit and (**p12**) the minimal steady-state frequency (F_threshold_). (**p13** and **p15**) Amplitudes of the first (A1) and second (A2) action potential (AP) were measured from the threshold to the positive peak on the first step, where at least 3 APs were induced. (**p14** and **p16**) AP durations were measured at half-amplitude (D1 and D2). (**p17**) The amplitude and (**p18**) duration of the maximal AHP was measured for the first AP. (**p19**) The amplitude and (**p20**) duration of the maximal afterdepolarization (ADP) was measured for the first AP. (**p21**) Amplitude variation (Var A) and (**p22**) duration variation (Var D) were computed as (A1 − A2) / A1 * 1,000 and (D2 − D1) / D1 * 1,000, respectively. At higher stimulation intensities, the maximal firing rate was defined as the last trace before the prominent reduction of the action potential amplitude, indicative of a saturated discharge. (**p23**) Imax is the minimal current-inducing saturating frequencies. To take into account the biphasic spike frequency adaptation (early and late), instantaneous firing frequency was fitted to a single exponential with a sloping baseline, according to the equation F_sat_ = A_sat_ × E^−t/τsat^ + t × m_sat_ + F_max_, where (**p24**) A_sat_ corresponds to the amplitude of early frequency adaptation, (**p25**) τ_sat_ to the time constant of early adaptation, (**p26**) F_max_ the maximal steady-state frequency, and (**p27**) m_sat_ to the slope of late adaptation. During the 800 ms depolarization protocol, a pronounced reduction of the amplitude of the action potentials was followed by an increase in the spike amplitude. This difference in action potential amplitude was termed the amplitude accommodative hump (**p28**).

Miniature postsynaptic currents were recorded in voltage-clamp mode at a holding potential of −60 mV, which was less negative than the reversal potential for mIPSCs. The pipette solution contained a low concentration of Cl (1 mM), resulting in an ECl of −111 mV. Therefore, inward currents were considered as mEPSCs and outward currents as mIPSCs [42]. Miniature events were recorded by including tetrodotoxin (TTX, 1 μM, Sigma) in the aCSF. IV curves were performed in voltage-clamp by applying voltage step of 800 ms from −200 to 50 mV. During 10 min, the biocytin diffused through the astrocytic gap junction network. Intrinsic neuronal properties were assessed in current-clamp by applying current steps (800 ms) from −100 pA until firing saturation, in 10 pA increments. Data were acquired using a MultiClamp700B (Axon Instruments) amplifier connected to an acquisition board (Digidata 1440; Axon Instruments) attached to a computer running the pCLAMP software (Axon Instruments). They were filtered at 2 kHz, digitized at 10 kHz. All electrophysiological recordings were analyzed using Clampfit and Igor.

### Drugs

TTX (1 μM, Sigma) and XE-991 dihydrochloride (10 μM; Sigma) were applied 15 min prior recordings and then throughout recordings. γ-DGG (0.5 mM, Tocris) and CPP (10 μM, Tocris) were used to assess glutamate level at synapses.

### Immunohistochemistry

Mice were deeply anesthetized with Dolethal (Pentobarbital) and perfused intracardially with PFA (2%) 2 weeks after AAV injection. The brains were carefully dissected and postfixed overnight with PFA 2% at 4°C. The next day, PFA was removed and the brains were cryoprotected overnight in PBS containing 30% sucrose. The brains were then cut into 40 μm sections with a freezing microtome (Leica, Germany). Slices were stored in PBS at 4°C until use. For immunohistochemistry, slices were blocked with PBS-Gelatin-Triton (PBS with 0.2% of gelatin and 0.25% of Triton), for 1 h at room temperature, incubated overnight at 4°C with primary antibodies (S100β: at 1:500, GFP: Abcam, chicken, ab13970 at 1:500, Cx30: Life Technologies, Rabbit, 712200 at 1:500) in the blocking solution, then washed 1 h at room temperature with PBS-Gelatin-Triton, and incubated 2 h with secondary antibodies (Streptavidin: Molecular Probes, 1:200, goat anti-chicken 488: 1:2,000, goat anti-mouse 555: 1:2,000) and DAPI (1:1,000) in the blocking solution and washed 3 times with PBS before mounting with Fluoromount (Invitrogen). Z-stack images were acquired at a confocal microscope with a 63× magnification with the same laser intensity, gain, and offset parameters (Leica SP5 inverted confocal) and then reconstructed using ImageJ software. GFP^+^ and S100β^+^ cells were counted manually, in the same number of slices in a Z-stack, in at least 1 region of interest (ROI) per slice per animal, and at least in 3 mice. Average fluorescence intensities for Cx30 were measured in hippocampal CA1 astrocytes in at least 3 nonoverlapping ROI per slice, 3 slices per animal, and 3 mice injected with AAV-GFAP-GFP or AAV-GFAP-Cx30 and were then normalized to AAV-GFAP-GFP expression. Isolated astrocytes were selected based on their GFP staining. After biocytin immunolabeling, Z-stack images were acquired at a confocal microscope with a 20× magnification (Leica SP5 inverted confocal). With ImageJ software, the number of labeled astrocytes was counted to determine the size of the astroglial network.

### iDISCO

Mice were deeply anesthetized with Dolethal (Pentobarbital) and perfused intracardially with PFA (4%) 2 weeks after AAV injections. The brains were carefully dissected and postfixed overnight with PFA 4% at 4°C. We used the iDISCO protocol [43] to quantify the volume of AAV injections in the hippocampus. Fixed samples were dehydrated progressively in methanol/PBS, 20%, 40%, 60%, 80%, and 100% for 1 h each. They were then incubated overnight in a solution of methanol 33%/dichloromethane 66% (DCM) (Sigma 270997). After 2 × 1 h washes with methanol 100%, samples were bleached with 5% H_2_O_2_ in methanol at 4°C overnight. After bleaching, samples were rehydrated in methanol for 1 h each, 80%, 60%, 40%, 20%, and PBS. Samples were washed rapidly with PBS and then incubated 2 × 1 h in PTx2 (PBS/0.2% Triton X-100). Pretreated samples were incubated in permeabilization solution (8% PBS/0.16% Triton X-100/20% DMSO/Glycine 23 mg.mL^−1^) at 37°C for 48 h and then incubated in Blocking Solution (8.4% PBS/0.168% Triton X-100/10% DMSO/6% NGS) at 37°C for 48 h. Samples were incubated in primary antibody dilutions in PTwH (10% PBS/0.2% Tween-20/0.1% heparin)/5% DMSO/3% NGS at 37°C for 7 days. Samples were washed 5 times in PTwH until the next day and then incubated in secondary antibody dilutions in PTwH/3% NGS at 37°C for 6 days. Samples were finally washed in PTwH 5 times until the next day before clearing and imaging. After immunolabeling, samples were dehydrated progressively in methanol in PBS, 20%, 40%, 60%, 80%, and 100% each for 1 h. They were then incubated overnight in a solution of methanol 33%/DCM 66%, followed by incubation in 100% DCM for 2 × 15 min to wash the methanol. Finally, samples were incubated in dibenzyl ether (DBE) (without shaking) until cleared (4 h) and then stored in DBE at room temperature before imaging. Brains were imaged with a light sheet Ultra Microscope II Lavision-BioTec. The volume reconstruction was performed with Imaris software.

### Novel object recognition test

Mice were injected bilaterally in the hippocampi with AAV-GFAP-GFP or AAV-GFAP-Cx30 at P21. After 3 weeks, mice were exposed to the NOR test [44]. NOR apparatus consisted of a dark-gray polypropylene box (40 × 30 × 23 cm, length, width, height). A glass cube (5 × 5 × 5 cm) and a ceramic bowl (8 cm in diameter, 5 cm in height) were used for object recognition. Objects were too heavy to be displaced by the animal and were positioned at 2 corners of the apparatus. The mice did not show any preference for either of the 2 objects. Behavioral tests were conducted during the light phase of the 12-h light/dark cycle under dim illumination (50 lux), between Zeitgeber time (ZT)-7 and ZT-9, so between 7 and 9 hours after the light was switched on in the animal house, and recorded using LifeCam Studio camera and software (Microsoft). Before the first NOR test, for 3 consecutive days, animals were handled for 2 min per day. On the day preceding each NOR test, animals were allowed to freely explore the empty (without objects) apparatus for 10 min for habituation. The NOR test consisted of 2 trials (T1 and T2) separated by an intertrial time interval (ITI). On T1 (acquisition trial), subjects were placed in the apparatus containing 2 identical objects for 20 min before being returned to their home cage for an ITI of 24 h. Then, they were placed back in the NOR apparatus containing a familiar and a novel object for 10 min (T2, restitution trial). The type (familiar or novel) and the position (left or right) of the 2 objects were counterbalanced and randomized within each experimental group during T2. Between each trial, the NOR apparatus was cleaned with water and the objects with 70% ethanol. Exploration was defined as the animal directing the nose within 0.5 cm of the object while looking at, sniffing or touching it, excluding accidental contact with it (backing into, standing on the object…). The raw exploration data were normalized to the total object exploration time.

### Statistical analysis

All data are expressed as mean ± SEM. Prior statistical comparison, normality tests, as well as variance analysis were performed, and the appropriate two-sided parametric or nonparametric statistical test was used. Two-tailed unpaired was used for between-group comparisons. Statistical significance for within-group comparisons was determined by one-way or two-way ANOVAs followed by post hoc tests. The Kolmogorov–Smirnov test was used for cumulative distribution comparison. Statistical analysis was performed using Prism (GraphPad software, version 8, CA, USA).

## Supporting information

S1 Data Supplementary data containing all the individual numerical values. (XLSX)

## Abbreviations

AAV: adeno-associated vector
aCSF: artificial cerebrospinal fluid
ADP: afterdepolarization
AHP: afterhyperpolarization
AP: action potential
CPP: 3-(RS)-(2-carboxypiperazin-4-yl)-propyl-1-phosphonic acid
Cx: connexin
DBE: dibenzyl ether
DCM: dichloromethane
fEPSP: field excitatory postsynaptic potential
ITI: intertrial time interval
ITR: inverted terminal repeat
LTP: long-term potentiation
mEPSC: miniature excitatory postsynaptic current
mGluR: metabotropic glutamate receptor
NMDA: N-methyl-d-aspartate
NOR: novel object recognition
PFA: paraformaldehyde
PPF: paired pulse facilitation
PTP: posttetanic potentiation
ROI: region of interest
TTX: tetrodotoxin
ZT: Zeitgeber time
γ-DGG: γ-D-glutamylglycine
